# Flood Risk Evaluation in Urban Spaces: The Study Case of Tormes River (Salamanca, Spain)

**DOI:** 10.3390/ijerph16010005

**Published:** 2018-12-20

**Authors:** Marco Criado, Antonio Martínez-Graña, Javier Sánchez San Román, Fernando Santos-Francés

**Affiliations:** 1 Department of Geology, Faculty of Sciences, University of Salamanca, 37008 Salamanca, Spain; marcocn@usal.es (M.C.); javisan@usal.es (J.S.S.R.); 2 Department of Soil Sciences, Faculty of Environmental Sciences, Avenue Filiberto Villalobos, 119, University of Salamanca, 37007 Salamanca, Spain; fsantos@usal.es

**Keywords:** flood hazard, urban vulnerability, urban exposure, probability, GIS

## Abstract

The expansion of cities towards flood zones, and the increasingly frequent episodes of torrential rains arising from global warming, mean that the population is becoming more exposed to floods. Due to this, a correct assessment of flood events is of great help in the development of preventive actions, planning and resource management, or interventions. For this reason, in this work we aim to establish guidelines to assess the hazard, exposure, and vulnerability of the population and its properties to flood events, using Hec-Ras for the simulation of the flood and ArcGis and GeoHecRas to treat geographic information and prepare the cartography. The study was focused on the Tormes River in Salamanca (Spain). We studied three return periods with different probabilities of occurrence and intensity, corresponding to 5, 100, and 500 years. The flow corresponding to each episode was calculated, along with the extension, speed, and depth that would be achieved in each case. Then, the probability of occurrence was delimited, as well as the magnitude, allowing us to obtain different hazard maps. In addition, the areas of greatest hazard to people and property were established for each event. Regarding the exposure, the areas and land use, infrastructure, and buildings that would be flooded in each case were identified, quantifying the extension or length of the affected properties at the different levels of hazard in each case. Additionally, the vulnerability of the different buildings and exposed infrastructure was studied. Finally, the flood risk was estimated by combining these three components.

## 1. Introduction

The world population is increasingly gathering in large urban environments [1,2], and in 2050 it is estimated that 68% of the world population will be concentrated in urban agglomerations, compared to the current 55% [3]. Given this fact, it is vital to incorporate analysis of the environment and natural risks in resource planning and territorial planning, especially when defining new areas that may be urbanized, in order to guarantee greater protection for the population and their properties [4,5,6,7,8].

According to the Center for Research on the Epidemiology of Disasters (CRED) and the United Nations Office for Disaster Risk Reduction (UNISD) in the period between 1998–2017, floods were the events that caused the greatest number of disasters (3148 cases, accounting for 43.4% of registered disasters), affecting 2 billion people, of whom 142,088 died (11% of total deaths, after those caused by earthquakes, storms, and extreme temperatures). The economic losses of floods reached 65.600 billion dollars (23% of the total losses caused by natural disasters, after tropical storms and earthquakes). Fortunately, in contrast with an increasing number of events and economic losses, the trend in human deaths related to flooding indicates a decrease, mainly due to meteorological systems and alert systems [9].

Another aspect of the impact of floods on society to take into account is the increase in extreme weather events linked to global warming [10], which increase the virulence of floods and their hazard [11,12,13,14,15,16]. In addition, Jongman [17] predicted that in 2050 extreme flood events will occur more frequently, shifting from every 50 to every 30 years, while damage cycles will occur every 10 years instead of every 16 years. The related losses in Europe would go from 4.9 billion annually to 23.500 billion, an increase of 380%, due to climate change and GDP increase. However, if about 1.750 billion is invested in risk prevention and mitigation measures, by 2050 the losses could be reduced by 30% (approximately 7 billion). In contrast, in certain regions the risk of flooding will decrease due to global warming, such as in areas of Northeastern Europe, where it will cause less snow accumulation than occurs at present [18,19,20,21].

Due to urban expansion and the generalized invasion of flood plains [22,23], and the higher frequency of extreme events that cause floods [24], it is important to study and simulate floods in order to mitigate the damage they cause [25,26,27]. The damage can affect people, causing human losses, increased risk of spreading diseases, eviction of homes, drownings, injuries, and other problems [28,29,30]; or it may result in economic losses related to basic services—electricity, water, telephone, or the Internet—or infrastructure, such as the destruction and/or flooding of roads and bridges, deterioration of homes, or destruction of crops [31,32,33,34]. In relation to this, the impacts caused by floods on water supply and evacuation networks are important, due to which several authors have developed methods to analyze these risks [35,36] with which the safety and reliability of these networks has been improved [37].

Generally, a flood consists of the overflow of a river outside its natural course, causing the temporary flooding of the surrounding lands [38]. There are different types depending on the agents that originate it. These may be natural, such as periods of heavy rains or snowmelt, or be caused by derivatives of human action, such as the breakage of dams, malfunction of dams, or damming or deforestation of large areas. In these latter cases the flood is considered a technological risk, and not natural. Broadly speaking, flooding may be slow and progressive, characteristic of prolonged periods of intense rainfall in watersheds, or involve a sudden and rapid flow, characteristic of episodes of torrential rains in short periods of time, which mainly affect small and steep basins.

The risk of flooding corresponds to the set of losses (human, economic, ecological, and so forth) that are expected due to the occurrence of a flood episode. The risk analysis covers three phases: risk factors analysis (on which this work focuses); risk assessment (in which the losses are estimated); and analysis and design of risk mitigation measures. In relation to the risk factors, they include the hazard, exposure, and vulnerability, and the conjunction of these factors simultaneously is the overall risk [39]. These risk factors have been widely used by various authors to estimate flood risk and related losses [40,41,42,43,44,45].

The hazard is related to magnitude (susceptibility to risk from the conditioning or passive factors such as the topography or geological substrate) and the severity and frequency with which the agent that causes the risk is expressed (probability of triggering factors). In the case of floods, the hazardousness corresponds to the depth, speed, and extension reached by the sheet of water in each event. Therefore, to determine the hazardousness, we analyze the magnitude and probability of the occurrence of each event.

Exposure corresponds to the properties (human, material, ecological, economic, and so forth) that can be damaged by the action of the danger according to its location (elements at risk). These properties may vary over time and, currently, due to economic development, population growth, and climate change, they are increasing [46]. We only estimate how vulnerable these elements are according to their location, and in the case of floods, it depends mainly on the distance to the channel from where the element is located. On the other hand, vulnerability refers to the expected loss (which could be material or social) of a specific element exposed to the risk—in this case, to the flood—and depends on the intrinsic or specific characteristics of each exposed element [47,48,49]. Based on these considerations, the possible mitigation measures existing in each case can be taken into account.

For these reasons, the objective of this work is to analyze the existing flood risk in an urban environment for events of different magnitude and probability. In this case, in Salamanca, a World Heritage Site with many historical infrastructures of great value in the river environment (a Roman bridge, churches, convents, and monasteries). On the one hand, this will allow delimiting of the areas, infrastructures, and buildings that could be affected by these floods, and the hazard, exposure, and vulnerability in each zone. It will be possible to use this information to delimit the unfit areas to be urbanized due to the risk of flooding, and to elaborate preventive measures or plans of action in case of flooding of the elements classified as vulnerable. On the other hand, the lacking studies of the flood risks in this city mean that the urban development and infrastructures on the floodplains have been partially limited. This work can help urban managers in the initial stages of urban planning when defining new urbanization areas that do not entail risk for the population.

To achieve these objectives, we propose an easy and low-cost method that allows integrating in its different phases a large part of the parameters involved in floods. The data are integrated in a Geographical Information System (GIS) and computed with Hec-GeoRas (Hydrologic Engineering Center of US Army Corps of Engineers, USA), which together with the use of LIDAR Digital Elevation Model (DEM) data of 1 m resolution, making it a more accurate method that will optimize the flood mapping.

The use of Hec-Ras (Hydrologic Engineering Center of US Army Corps of Engineers, USA) for flood´s modeling and the design of flood mapping with ArcGis (ESRI, USA) has been widely used in the study and modeling of floods. The use of Hec-GeoRas and the increasingly use of greater and greater precise DEM have allowed to improve the accuracy of this type simulations, so the information available for flood management has improved [50,51,52,53]. However, several authors criticize the effectiveness of flood mapping in flood assessment.

In the last few years, some authors have developed simulation models of floods with diverse mathematical basis, which aim to solve the limitations of traditional models, as well as to seek the adaptation of models to specific time-space cases. Nowadays, there are a great diversity of models (mainly, empirical methods, based in observations; hydrodynamic models, mathematical models that attempt to replicate fluid motion, and depending on their spatial representation of the floodplain flow, the models can be dimensionally grouped into 1D, 2D, and 3D models; and simplified methods, non-physics-based) used in flood risk mapping, flood damage assessment, real-time flood forecasting, flood related engineering, water resources planning, river bank erosion, and floodplain sediment transport, contaminant transport, floodplain ecology, river system hydrology, or catchment hydrology [54]. Additionally, many authors have focused their efforts on identifying the impact that different parameters—such as roughness or vegetation—have on the water dynamics in floods [55,56].

### Description of the Study Area

**Figure 1:**
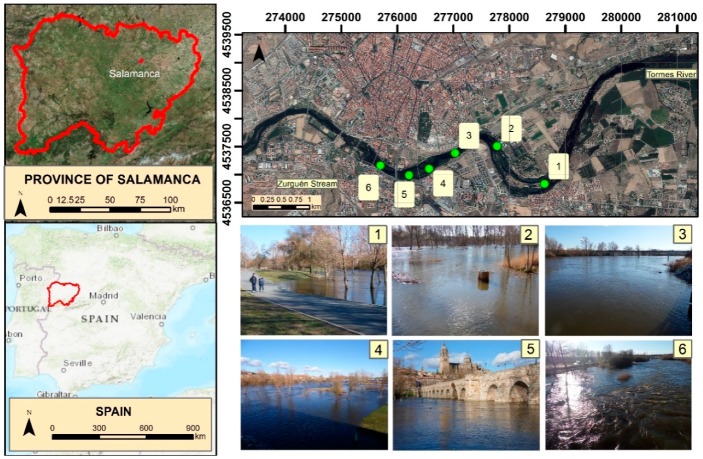
Study area and Tormes River in: (1) Fluvial island, in Santa Marta de Tormes; (2) “La Aldehuela”, in Cabrerizos; (3) Next to the water treatment plant of Salamanca; (4) Upstream of the Roman bridge; (5) Close to Roman Bridge and the Cathedrals; (6) Beside the hospital and the “Salas Bajas” sport center.

This study focuses on the Tormes River as it passes through Salamanca and its surroundings, as well as on a small tributary (Zurguen stream) that pours its waters into the Tormes in this city (Figure 1). The Tormes River divides the city of Salamanca into two halves, and determines the spatial configuration of the city and outskirts, bringing almost 200,000 inhabitants together. The Tormes is born in the Mountain of Gredos and travels 150 kilometers before reaching Salamanca, draining into a basin of 4132 km^2^ at this point. Historically, the Tormes has been the protagonist of multiple episodes of flooding, having recorded 35 historical floods between the XII–XIX centuries [57]. In the middle of the 20th century the Santa Teresa reservoir, which regulates the flow of the Tormes, was built upstream from Salamanca, and the virulence of the floods decreased. However, the subsequent expansion of the city of Salamanca along the alluvial plains of the Tormes, which has been done without detailed flood studies, makes it a good case for the study of floods.

## 2. Materials and Methods 

The periods of return studied (T) correspond to 5 (T5), 100 (T100), and 500 (T500) years. The T5 corresponds to episodes of high probability and low magnitude, the T100 with episodes of medium probability and intensity, and the T500 with events of low probability but virulence.

To simulate the floods in each scenario, we differentiated two stages: the hydrological phase and the hydraulic phase. In the first, the maximum flows that exist for each T were estimated, and in the second, the flood was modeled, and we obtained the evolution of the water sheet along the process.

For the study of design flows, we divided the study into three sections: the Zurguén section, upper section of the Tormes, and lower section of the Tormes (after merging with Zurguén). The Tormes design flows were obtained from a historical series of maximum annual flows recorded in the Salamanca gauging station from 1979–2014 (Table 1). These data were treated using the Gumbel method, which determines the maximum flow rate for each T as a function of the mean and standard deviation of the flows obtained in the series. On the other hand, for the calculation of the maximum flow rates of the Zurguén stream, due to the absence of gauging stations an indirect estimation based on the rational method was employed that uses as parameters the maximum daily average rainfall for a certain T, intensity of precipitation, soil runoff coefficient, and surface of the basin. 

For the hydraulic phase, the channel of the rivers was first modeled (the main channel, banks, and alluvial plain were delimited). For this, ArcGis 10.5 and the extension Hec-GeoRas, and a 1 × 1 DEM and an aerial image as a base supplied by the National Geographic Institute of Spain, were used. Subsequently, the channel typology was exported to the Hec-Ras program, which simulates the flood. The Hec-Ras program, based on the maximum flow rates obtained for each scenario, and the configuration of the channel, simulated the extension, depth, and speed that the volume of water would present in each place in the different T scenarios. Finally, this information was exported again to ArcGis 10.5, and the corresponding cartography was elaborated, related to the extension, depth, and speed that the water presents in each scenario.

Concerning to the analysis of risk factors, one was carried out for each scenario (T5, T100, and T500). This way, the different hazard levels, exposure, and vulnerability for each of the return periods were analyzed. The magnitude was obtained as a result of combining the depth and speed reached by the water sheet in each case. The magnitude, together with the probability, determines the hazard. The exposure was determined by delimiting the buildings, areas, or infrastructure affected by the flooding of each scenario. The vulnerability was determined through a field campaign studying the characteristics of each exposed element.

In relation to the magnitude, the speed and depth raster layers obtained after the analysis with the Hec-Ras and Hec-GeoRas program were reclassified and three types of intensity identified: low (1), medium (2), and high (3) (Table 2). Then, the rasters are multiplied (velocity: 1, 2, 3 × depth: 1, 2, 3), and the results (1,2,3,4,6,9) are reclassified into levels of high (9), medium (4,6), and low magnitude (1,2,3). For the elaboration of the hazard map, the possible affectation to properties was distinguished, which corresponded to the three levels of magnitude established. Low hazard areas do not generally involve property losses, with only small objects and vehicles being vulnerable. The zones of medium hazard can expect an impact on weak structure buildings. In areas of high hazard, any type of construction may be affected. In addition, hazard maps include areas that present a hazard to people based on the speed and depth of the water. These danger zones for people are those with depths greater than one meter or with water velocity greater than 1 m/s, and also those that present with a product depth × speed greater than 0.50. 

In the context of the elements present in the river environment, first of all, a classification of land uses was obtained from the entire study sector of the Soil Occupation Information System of Spain. Then, this classification was refined with orthoimages and field work, to better specify the elements exposed to the different scenarios.

After this, the exposure of the elements present in the river banks was analyzed (Table 3). To do this, the hazard mapping for each return period was superimposed on the cartography of land uses and exposed elements. In this way, the areas of land, infrastructure, and buildings that could be affected by each event with a high, medium, or low magnitude were identified, giving rise to high, medium, or low exposures, respectively. Next, we estimated the area of each type of land use that was flooded in each episode. Infrastructure and buildings affected by water were digitized in vector files, after which the length of infrastructure and the number of buildings that could be affected in each scenario were estimated.

After assessing the exposure, the vulnerability of these elements identified as exposed to the flood was analyzed. To insert the value of the vulnerability in the risk analysis, the different elements exposed to the flood were grouped according to their constructive characteristics, after being reclassified according to their high, medium, or low vulnerability (Table 3).

Finally, the flood risk of the Tormes as it passes through Salamanca was estimated. To estimate the final risk, different levels of hazard, exposure and vulnerability were reclassified (High = 3, Medium = 2, Low = 1). Subsequently, these three parameters, and taking into account the defined intervals, are combined according to the risk estimation formula [59] (Equation 1). As a result of the multiplication, values of 1, 2, 3, 4, 6, 8, 9, 12, 18, and 27 units are obtained, which are reclassified, giving rise to flood risk categories: very low (1 and 2 units), low (3 and 4), medium (6 and 8), high (9 and 12), and very high (18 and 27).

Risk = Hazard × Exposure × Vulnerability(1)

## 3. Results and Discussion

### 3.1. Design of Flood Events

**Figure 2:**
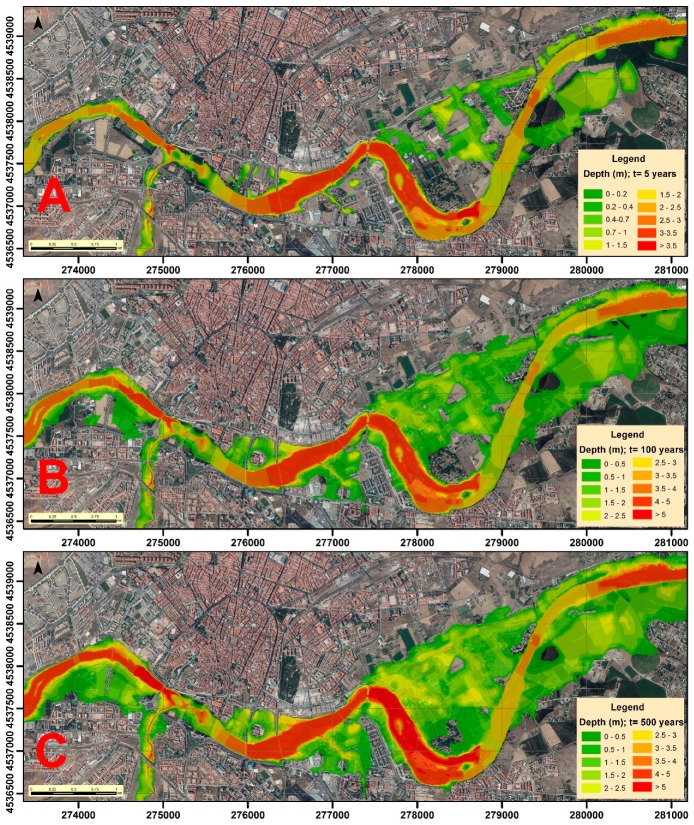
Water depth (m) in the different scenarios: (**A**) T = 5 years; (**B**) T = 100 years; and (**C**) T = 500 years.

**Figure 3:**
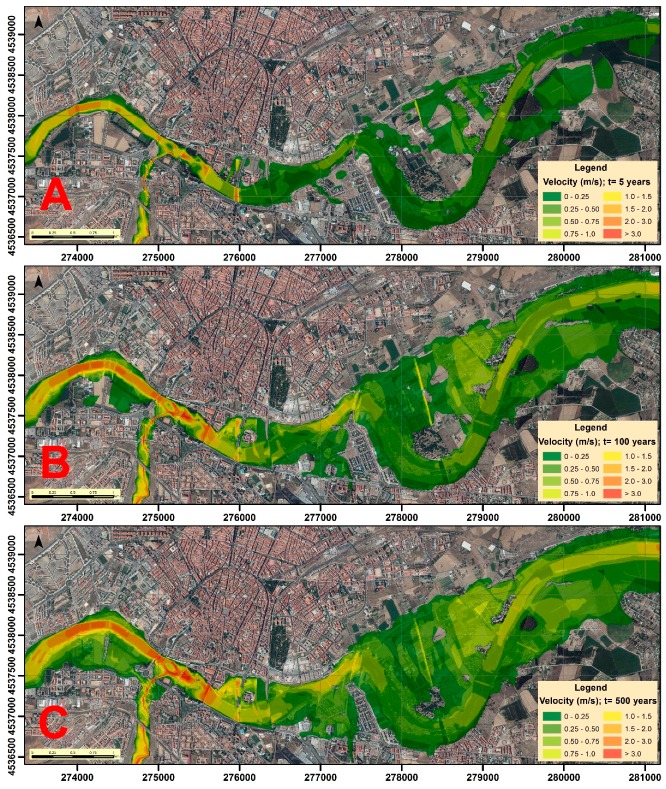
Water velocity (m/s) in the different scenarios: (**A**) T = 5 years; (**B**) T = 100 years; and (**C**) T = 500 years.

In order to model the floods in each scenario, the extension, depth and velocity of the volume of water were determined with Hec-Ras. Previously, the corresponding return flows that we estimated were inserted (Table 4). Hec-Ras simulates the transition of these flows through the channel, and the resulting information was processed with ArcMap 10.5, allowing the depth (Figure 2) and velocity (Figure 3) cartographies of the water sheet for the return periods of 5, 100, and 500 years to be obtained. Obviously, the greatest depths will be reached in the areas corresponding to the channel of the river, similar to what happens with speed, although this may vary according to the depth and type of surface (roughness of the ground) over which the water flows in every moment.

### 3.2. Probability and Magnitude

**Figure 4:**
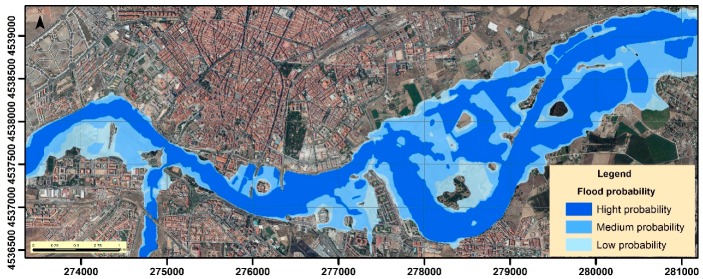
Flood probability of the areas surrounding the river.

The flood probability of the different zones surrounding the Tormes channel depends on the magnitude of the event in question. The banks and spaces near the river present higher probabilities, while in the furthest zones the probability is lower, due to requiring the occurrence of a high magnitude event (Figure 4).

**Figure 5:**
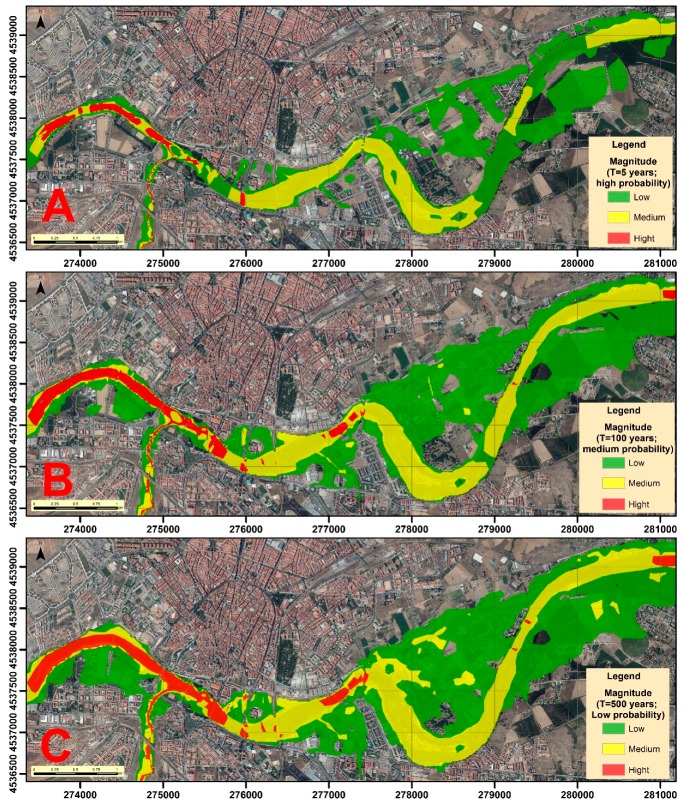
Magnitude of floods for each return period: (**A**) T = 5 years; (**B**) T = 100 years; and (**C**) T = 500 years.

The magnitude (Figure 5) is always higher in the areas near the channel, considering that the depth and velocity are higher. The magnitude is lower in the floodplains, although in the events with a greater return period it is observed that the zones of high and medium magnitude increase and expand towards them.

### 3.3. Hazard Map

**Figure 6:**
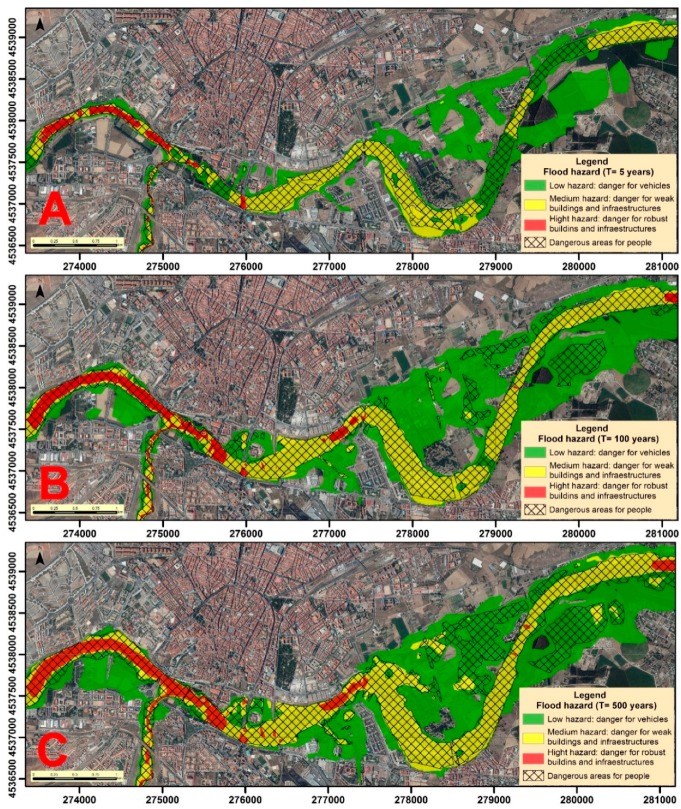
Hazard maps for each return period: (**A**) T = 5 years; (**B**) T = 100 years; and (**C**) T = 500 years

Hazard maps are developed from the magnitudes and probabilities studied in each return period (Figure 6). These maps indicate the existing hazard in each scenario for the people and the different properties. The risk to people is delimited by a mesh that indicates that the depth and speed of water at these points could cause a person to drown. The impact on properties is divided into three intervals: low, which entails danger for vehicles and light elements; medium, where weak and unstable constructions could succumb; and high, where all buildings and infrastructure could be damaged.

In relation to hazard zones for people, although this may vary according to the age and physical capabilities of each person, they are limited mainly to areas near the riverbed, and are scarce in the alluvial plains except in the events of greater magnitude. Regarding the danger for material goods, we can also see how the areas of impact are greater when the magnitude is greater and the probability is lower. The greatest hazard occurs in areas adjacent to the channel, being medium, or low in areas of the alluvial plain, due to the amplitude of the same that allows expansion of the water surface.

### 3.4. Exposure of Elements

The different impact (high, medium, and low) of future floods on the elements that constitute the urban environment, according to their location, was evaluated. We studied the extent of the areas that will be flooded in each episode, and to what land use these areas correspond, as well as the infrastructure and buildings affected.

**Figure 7:**
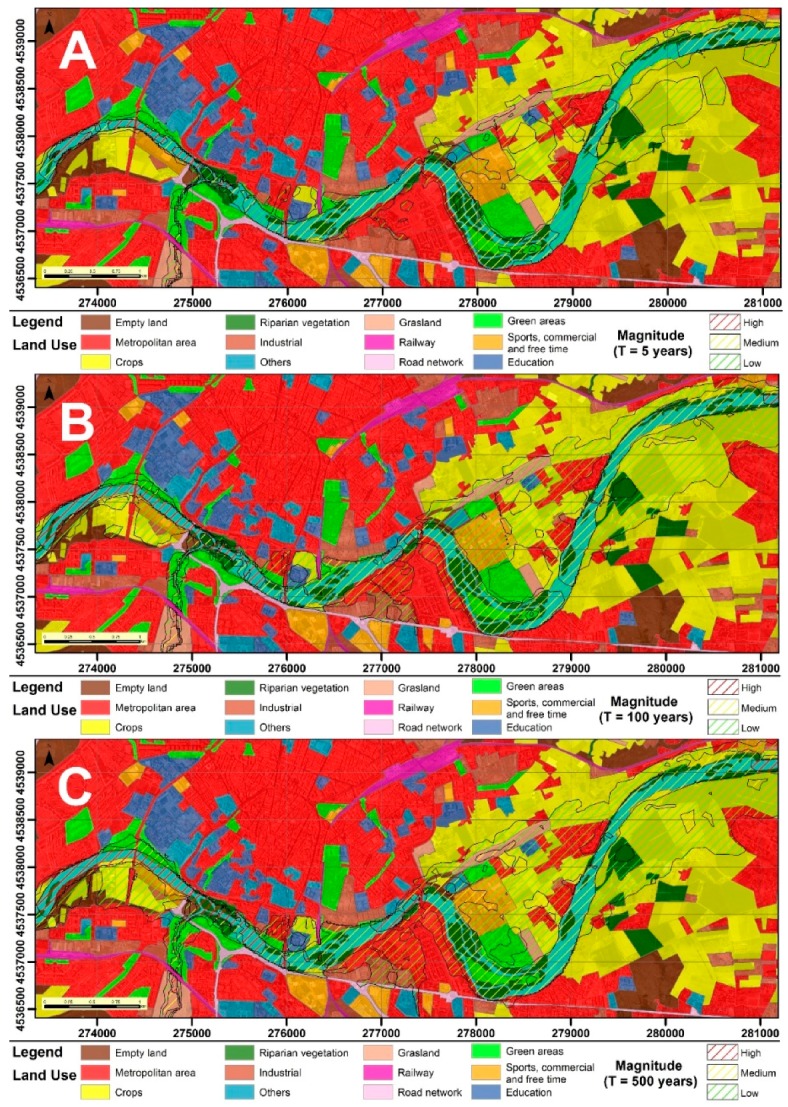
Flooded areas in each scenario according to their land use: (**A**) T = 5 years; (**B**) T = 100 years; and (**C**) T = 500 years.

In the cartography of land uses affected by the floods in each scenario (Figure 7), we can observe the different land uses exposed to high, medium, and low hazards, whose extension was calculated (Table 5). The main flooded areas correspond in all cases with agricultural areas, mainly intended for irrigated crops. The areas that support buildings are also quite affected, with up to 100 Ha of urban land being impacted in an event of greater magnitude. Green areas, areas with riparian vegetation, and grasslands are also affected, while the rest of the land use types are only slightly affected.

**Figure 8:**
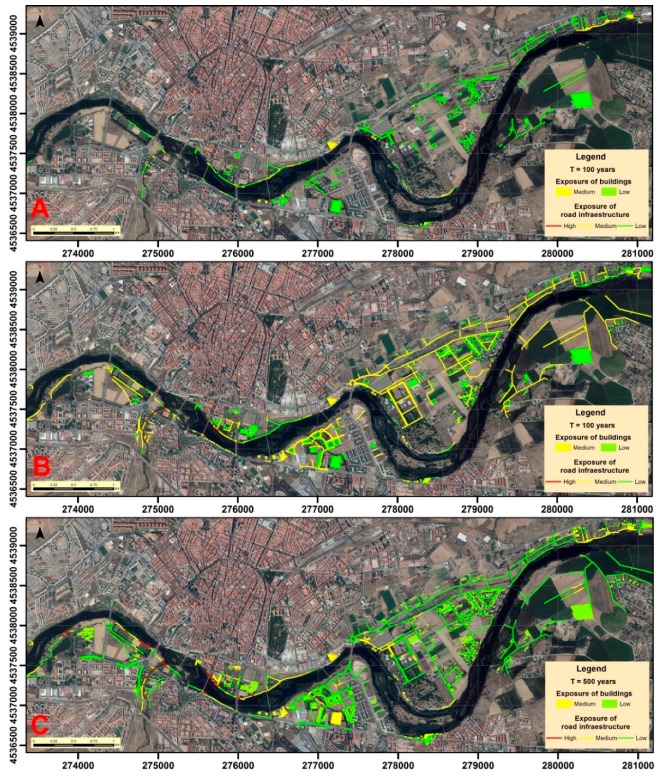
Exposure of the elements identified in the different return periods: (**A**) T = 5 years; (**B**) T = 100 years; and (**C**) T = 500 years.

On the other hand, the infrastructure and building exposition related to each scenario (Figure 8) was quantified when determining the length of affected infrastructure sections (Table 6), and when counting the number of vulnerable building types (Table 7). In terms of communication routes, roads are the most affected as a whole, however, among bike lanes and unpaved roads, the percentage of sections affected by high and medium exposures is higher, due to their predominance in areas close to the Tormes. Regarding the different types of buildings, none were identified in the high exposure areas. However, they are frequent in areas of medium and low exposure, especially buildings located in urbanizations of a certain purchasing power, corresponding mainly to duplex and detached houses. In areas near the river single-family homes are also common, along with vegetable patches. Flats and educational or industrial buildings are rare. On the other hand, buildings related to agriculture are very frequent. These include greenhouses, tool sheds in areas of kitchen gardens, and agricultural ships to house machinery. Buildings classified as "Other" generally refer to abandoned properties, or buildings that have other characteristics that do not allow them to be classified in another category because they are not representative.

### 3.5. Vulnerability of Elements

**Figure 9:**
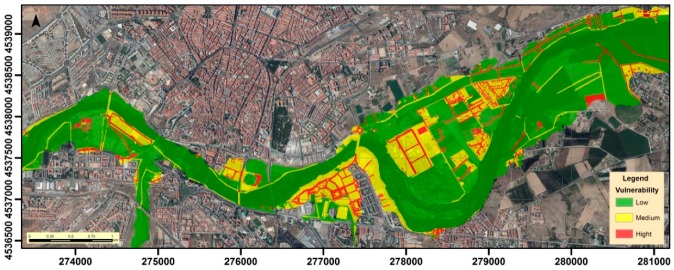
Vulnerability of the elements exposed to floods.

Vulnerability changes depending on the characteristics of each type of building, so the most vulnerable buildings are those designed to house agricultural tools and all buildings related to agricultural and livestock uses. The rest of the buildings could be considered low–medium vulnerability, due to their more robust structure, but because they tend to be in places with frequent human presence, they are considered of medium vulnerability (Figure 9).

### 3.6. Flood Risk

**Figure 10:**
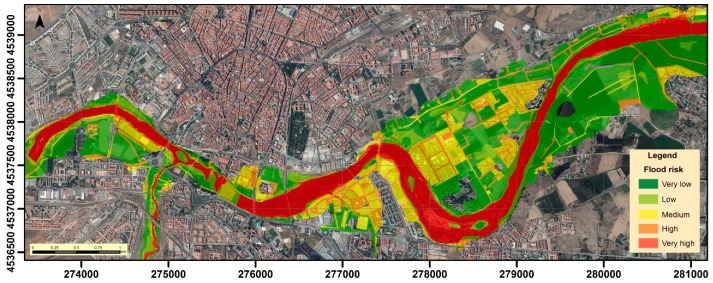
Flood risk estimated in the study area.

Finally, regarding the risk of final flooding (Figure 10), the areas of greatest risk (very high risk) cover an area of 160.3 Ha, which correspond to those areas of greatest hazard, exposure and vulnerability, and are mostly linked to the Tormes channel and closer areas. At high risk, we find those elements most exposed and vulnerable to flood, including some communication routes and most buildings, accounting for an extension of 57.9 Ha. Medium risk covers an area of 124.2 Ha and includes the rest of the exposed elements, especially communication routes and the areas surrounding them and those extensions whose main land use is to house buildings, infrastructure, and services of different types. The medium risk zones corresponds to areas of medium or high exposure and vulnerability, but of low hazard, or with highly hazardous areas, but low exposure and vulnerability. With low and very low risk of flooding, the rest of the extensions (covering areas of 72.2 Ha and 278.9 Ha, respectively) appear, which correspond to areas of the Tormes floodplain, are generally far from the river, and in which agricultural activities are developed, although there are also extensions with pastures, riparian vegetation, or empty lands.

### 3.7. Discussion

The obtained results show the impacts that will occur in the Tormes floodplain as it passes through the historic city of Salamanca in the different flood scenarios. With the risk mapping, first of all, a better management of the land uses can be carried out in this riversite, which is related to two concepts: (1) greater ease when managers limiting certain activities or uses in those exposed areas to the risk, although depending on the different type of risk, activities or uses compatible with each risk can be developed; and (2) greater security when urban planners allocating land uses to those areas of the riverbank that are not affected by the risk. Flood cartography has been presented as maps, which has been criticized by some authors, who affirm the inefficiency of this cartography due to it is not estimated by the planners. Some authors suggest that flood information should be presented on real and concrete 2D and 3D images, in a way that is more attractive and realistic [60], facts that can be achieved with this model, since with the generated layers and the ArcGis 2D images, 3D images, and flights can be design. In relation to this, in this work, the great extension of the study area acted in a limiting way when making this type of images, since only a part of that area could be represented. For this reason, numerous authors carry out studies in specific areas, using very large scales, improving even more the levels of precision [61,62,63]. Others authors maintain that the use of the flood information by governments and planers is due more to how information is exchanged with the planners than to the quality of the work [64], which is why some authors propose interactive work sessions of researchers and planners [65]. Other authors advise to integrate in the analysis other characteristics such as the demands of the social groups affected [66], an aspect that the proposed methodology does not agglutinate.

Regarding the method, although it is based on classic procedures, but of recognized solvency, it includes new elements that allow improving the results. This is the case of the Hec-Ras program, which is implemented in the GIS, in the form of Hec-GeoRas, so that the topographic description of flooded areas is precise, due to the 1 m resolution LIDAR DEM use [67]. Due to these DEM´s precision, the accuracy of these models increases [68,69]. For the case of the estimation of return flows, two procedures are proposed to obtain it, which depends on whether there is a gauging station. In addition, the different artificial barriers existing in the river (as in the case of dykes or bridges) are implemented in the model, in such a way that it increases the sensitivity of the model. Additionally, this infrastructure implementation evaluation can be used to study the possible impact on floods that an infrastructure of a project wants to perform in a certain floodplain. Furthermore, it is possible to identify those infrastructures that are most exposed to risk and hazardousness too, and depending on this risk and its characteristics, preventive management measures can be designed.

In conclusion, the method can be used and adapted in different places and environments, since its variety of opportunities allows us to adapt it to any situation and spatial configuration. In this case, The Tormes River and the city of Salamanca were chosen, due it being a river with a large number of historical floods in a historical, artistic, and cultural city. Despite this, flood studies were very scarce and not very precise, which has led to an irregular and inconsistent development and soil occupation on the riverbank. We hope that with this work this scarcity will be corrected and the management of the banks of the Tormes in Salamanca will be more effective and safe for the population.

## 4. Conclusions

Natural risks cause great losses to society, both in human and socioeconomic terms, with floods being one of the risks that cause the greatest impact on society. Therefore, the procedure followed in this work is an essential tool to guarantee adequate protection of the population and correct management of territory and land use, which will also affect the proper maintenance of socio-economic resources.

The methodology used for the flood risk assessment is easy, fast, and cheap to apply, as well as being robust and precise in the topography description of flooded areas due to the use of the high resolution spatial information (1 m resolution LIDAR DEM) and the design and implementation in the model of all the anthropic elements located in the channel that affect the flood evolution. The topography and the estimation of the return flows allow us to simulate adequately the characteristics of the flood in each event, meaning the hazard of the event can be effectively evaluated. Regarding the exposure of different properties, the method allows resources to be saved by analyzing exposure for only those exposed to the hazard, pre-determined due to orthophoto and field work, and their vulnerability is also evaluated. In addition, a characteristic of this method is that it allows us to discern between hazard zones for people and hazard zones for material goods. Therefore, the method employed in this work is considered broad and valid, since it takes into account several factors involved in the risk analysis, and is not based on individualized analyses of each factor. Concerning the estimation of return flows, the use of volumetric flow reduces the error and increases the accuracy. Definitely, it is an ad hoc method, in which a multitude of case studies can be simulated due to the parameters and infrastructures that can be designed and taken into account in the procedure. Consequently, furthermore to being able to adapt the model to a multitude of scenarios, it allows the design of post-installation scenarios in the river environment of new elements that interfere with floods evolution, so that it can be used for preventive purposes.

By the Tormes River in Salamanca, there are many homes and a lot of infrastructure that could be exposed to floods because they are located in the floodplain of the river. For this reason, the employed methodology provides the basis and criteria so that authorities and governments can regulate land use and limit human activity in the floodplains through proper territorial planning. Also, the existence of this risk cartography will allow a better manage of those floodplain areas that are not affected by the risk of flooding. Due to LIDAR and ArcGis integration in the model, very creative information could be generated regarding the flood episodes, in order to achieve a greater impact on the receiver, as well as very concrete and real recreations. Moreover, the quantification of expected damages or losses for future flood events in different scenarios will be possible, and also enables the elaboration of action plans and risk mitigation measures in areas with exposed and vulnerable buildings and infrastructure.

## Figures and Tables

**Table 1:** Maximum annual instantaneous flow for each year of the series [58].

**Year**	79–80	80–81	81–82	82–83	83–84	84–85	85–86	86–87	87–88
**Q_m_**	41.7	17.38	118.7	55.3	138.9	138.9	109.4	98.7	126.6
**Year**	88–89	89–90	90–91	91–92	92–93	93–94	94–95	95–96	96–97
**Q_m_**	26.5	378.9	99.1	20.56	14.9	176.9	33.9	316.8	252.0
**Year**	97–98	98–99	99–00	00–01	01–02	02–03	03–04	04–05	05–06
**Q_m_**	527.9	22.0	98.3	387.0	46.5	444.0	157.0	43.9	66.5
**Year**	06–07	07–08	08–09	09–10	10–11	11–12	12–13	13–14	35 years
**Q_m_**	270.0	136.0	55.3	189.0	168.0	50.4	205.6	172	
**Annual Studied Flows Statistics**									
**Statistics**		**Mean**				**Standard dev.**			
		148.70				129.97			

**Table 2:** Classification criteria proposed by the authors to evaluate the magnitude.

Velocity (m/s)				Depth (m)			
Magnitude	Low	Medium	High	Magnitude	Low	Medium	High
**Speed**	< 1	1–2	> 2	**Depth**	< 0.5	0.5–2	> 2

**Table 3:** Types of exposed elements and vulnerability that they present.

Exposure	Types of exposed elements	Vulnerability
**Road infrastructures**	Roads	Medium
	Cycle lane and unpaved roads	High
**Buildings**	Flat, chalet, duplex, single family home, industrial, education, sports–free time and others	Medium
	Farming and Tool shed	High
**Land Use**	Riparian vegetation, green areas, grasslands, empty lands and others	Low
	Crops and industrial	Medium
	Metropolitan area, sports-free time and education	High

**Table 4:** Design flows (m^3^/s) for each section in the different return periods.

Return Periods (T, in years)	Section		
	Tormes I	Zurguén	Tormes II
T = 5	217.3	106.3	323.6
T = 100	635.2	220.1	855.3
T = 500	926.0	307.6	1233.6

**Table 5:** Land use and extension (Ha) of flood zones in each scenario.

Land Use	T = 5 years			T = 100 years			T = 500 years		
	High	Med	Low	High	Med	Low	High	Med	Low
**Metropolitan area**	0.46	4.51	27.56	0.78	10.20	65.52	1.62	14.83	86.70
**Crops**	0.02	1.78	115.8	0.77	8.68	198.1	1.72	22.13	220.7
**Riparian vegetation**	0.22	34.12	19.24	3.18	43.58	15.95	6.72	42.65	16.47
**Green areas**	0.85	9.81	14.86	2.80	15.80	23.34	3.71	20.59	28.03
**Grasslands**	0.57	1.42	11.90	1.13	3.34	15.07	1.61	6.20	16.46
**Empty lands**	0.01	0.36	4.52	0.75	2.11	10.67	2.39	4.96	11.66
**Industrial**	-	0.28	2.61	-	2.26	10.51	-	2.68	14.07
**Education**	-	-	2.05	-	0.01	4.44	0.02	0.25	4.95
**Sports-free time**	0.05	0.51	9.94	0.56	1.64	33.74	0.99	9.12	27.75
**Others**	-	0.33	2.10	0.01	0.50	3.70	0.02	2.24	3.43
**Total**	2.18	53.12	210.6	9.98	88.12	381.0	18.80	125.7	430.2
	265.9			479.1			574.7		

**Table 6:** Impact of floods (km) on different types of urban roads in each scenario.

Affection to Road Infrastructures	T = 5 years			T = 100 years			T = 500 years		
	High	Med	Low	High	Med	Low	High	Med	Low
**Roads**	0.01	0.10	6.22	0.06	0.70	18.04	0.14	2.16	24.65
**Cycle lane**	-	0.45	1.59	0.18	1.43	1.27	0.36	1.77	2.23
**Unpaved road**	-	0.99	4.96	0.02	1.52	13.96	0.30	3.07	14.79
**Total**	0.01	1.54	12.77	0.26	3.65	33.27	0.80	7.00	41.67
	14.32			37.18			49.47		

**Table 7:** Number of buildings of each type affected in the different scenarios.

Affection to Buildings	T = 5 years			T = 100 years			T = 500 years		
	High	Med	Low	High	Med	Low	High	Med	Low
**Flats**	-	-	-	-	-	2	-	-	8
**Detached house**	-	-	85	-	1	151	-	1	171
**Duplex**	-	1	11	-	1	57	-	2	96
**Single family home**	-	2	17	-	4	32	-	6	46
**Farming**	-	5	50	-	6	79	-	16	94
**Tool shed**	-	9	39	-	12	61	-	19	70
**Industrial**	-	-	3	-	1	8	-	3	12
**Education**	-	-	12	-	2	14	-	6	10
**Sports – free time**	-	3	19	-	7	25	-	16	22
**Others**	-	3	12	-	5	46	-	7	69
**Total**	-	23	248	-	39	475	-	76	598
	271			514			674		
